# Diagnosis-Specific Sickness Absence and Subsequent Common Mental Disorders: A Register-Linkage Cohort Study among Finnish Public Sector Employees

**DOI:** 10.3390/ijerph17030782

**Published:** 2020-01-27

**Authors:** Elina Mauramo, Tea Lallukka, Minna Mänty, Hilla Sumanen, Olli Pietiläinen, Eero Lahelma, Ossi Rahkonen

**Affiliations:** 1 Department of Public Health, University of Helsinki, P.O. Box 20, 00014 Helsinki, Finland; tea.lallukka@helsinki.fi (T.L.); minna.manty@helsinki.fi (M.M.); hilla.sumanen@helsinki.fi (H.S.); olli.k.pietilainen@helsinki.fi (O.P.); eero.lahelma@helsinki.fi (E.L.); ossi.rahkonen@helsinki.fi (O.R.); 2 Finnish Institute of Occupational Health, P.O. Box 18, 00032 Helsinki, Finland; 3 Unit of Health Care and Emergency Care, South-Eastern Finland University of Applied Sciences, 48220 Kotka, Finland

**Keywords:** employees, mental disorders, sickness absence, work ability

## Abstract

Sickness absence is associated with poor health outcomes, but little is known about its consequences for general mental health. This study examined the associations between diagnosis-specific sickness absence and subsequent common mental disorders (CMD). Register data on medically certified all-cause sickness absence and sickness absence due to mental disorders and musculoskeletal diseases from 2004–2007 were linked to the Helsinki Health Study 2007 and 2012 survey data on City of Helsinki employees in Finland (*N* = 3560). Using logistic regression and multinomial logistic regression, we analysed the associations between the total number of reimbursed sickness absence days in 2004-7 and CMD General Health Questionnaire 12) in 2007 and 2012 and CMD changes. Sickness absence due to mental disorders (age- and sex-adjusted odds ratio (OR)range: 2.16 to 2.93), musculoskeletal diseases (OR range: 2.79 to 2.93) and all-cause sickness absence (OR range: 1.48 to 3.20) were associated with CMD in 2007. In 2012, associations with lower ORs were observed. Associations were also found with changing and especially repeated (OR range: 1.49 to 3.40) CMD. The associations remained after adjusting for work-related covariates and health behaviours. Diagnosis-specific sickness absence showed persistent associations with subsequent CMD and their changes. Attention should be paid to both the short- and long-term consequences of sickness absence for employee mental health.

## 1. Introduction

Sickness absence has been associated with subsequent poor general health and functioning [1,2], repeated and even longer sickness absence spells [3,4,5,6], suicidal behaviour [7], disability retirement [8,9,10] and premature mortality [11,12,13]. However, data on employee health after periods of sickness absence are still limited in comparison to the amount of information available on the factors preceding and predicting sickness absence. General mental health and common mental disorders (CMD), which include, e.g., non-psychotic depressive and anxiety disorders, have been particularly rarely investigated among employees after sickness absence spells.

Multiple health- and work-related factors affect employee situations that lead to absence from work. These factors include employee perceptions of health, current and previous health status, health behaviours, working conditions and job satisfaction [14,15,16]. Sickness absence should be a way for the employee to recover from sickness and ensure an increased level of health and work ability [1]. However, this is not necessarily seen at the population level, as the risk of different diseases and disorders actually increase post-absence. Sickness absence spells of varying durations are common, and they cause employers and employees, as well as societies, considerable expenditures that are even greater when the absence leads to an employee’s declining health [14,17]. Thus, the effects and consequences of sickness absence warrant more detailed investigation. 

CMD are prevalent among employees, affecting 20–30% of the employed European adult population yearly [18,19,20]. These disorders may lead to poorer work ability, psychosocial functioning and quality of life among employees [21,22,23,24,25,26,27,28,29,30]. Previous studies have associated CMD with subsequent sickness absence [22,23,24,25,26,27,28,29,30]. However, only a few of these studies have been able to examine sickness absence due to different diagnostic causes, and have mostly been limited to all-cause absence and mental causes [22,29,30]. A British study showed recent and repeated CMD to predict sickness absence due to mental causes but not due to other causes [22], whereas another European study found associations with all-cause absence and absence due to mental disorders [30]. Studies on disability retirement have also shown CMD to predict all-cause disability retirement as well as retirement due to mental disorders and disability due to musculoskeletal diseases [24,31]. 

In our prior work, we have found CMD at different severity levels as well as changing and repeated CMD to be associated with subsequent self-certified short and medically certified long sickness absence spells as well as with diagnosis-specific sickness absence including sickness absence due to mental disorders and musculoskeletal diseases [25,32]. In addition, all-cause sickness absence and absence due to mental disorders were associated with poor mental health functioning in our cohort of midlife and ageing employees [1].

Thus, although there is evidence of associations between general mental health and subsequent sickness absence, evidence of employee mental health after sickness absence due to different diagnostic causes is still scarce. Longitudinal studies utilising comprehensive data on diagnosis-specific sickness absence in combination with well-validated data on CMD with repeated measurement are essential. Such studies would increase the understanding of the long-term mental health consequences of sickness absence due to different causes. The aim of this study was to respond to this shortage of evidence and to examine whether medically certified diagnosis-specific sickness absence due to the two most common diagnostic causes of sickness absence, i.e., mental disorders and musculoskeletal diseases, is associated with subsequent CMD and their changes between two time points in a Finnish cohort of midlife and ageing municipal employees.

## 2. Materials and Methods

### 2.1. Data

**Figure 1:**
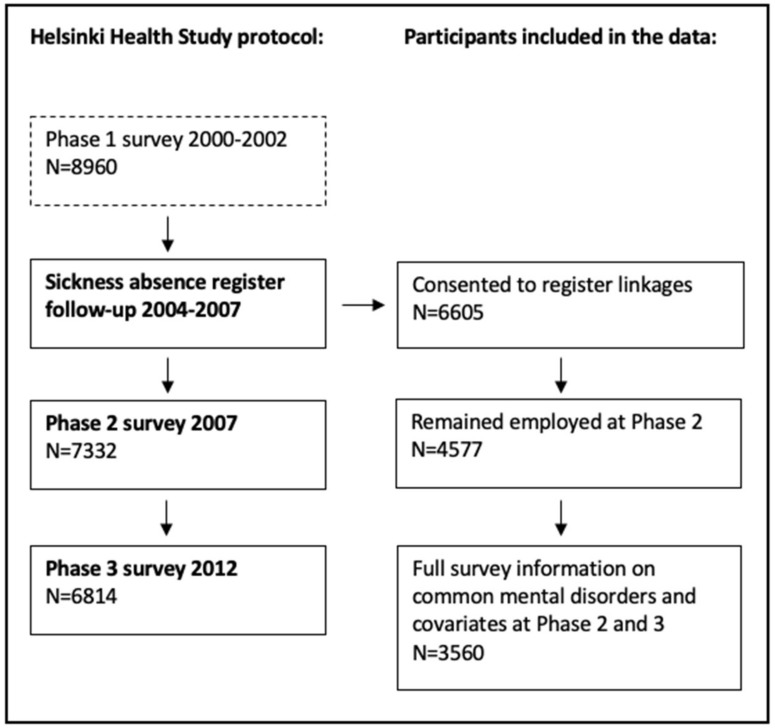
Flow chart of the data.

Survey data from the ongoing Helsinki Health Study (HHS) were linked with prospective administrative register data on diagnosis-specific and all-cause sickness absence from the Social Insurance Institution of Finland. The HHS Phase 1 postal surveys were conducted in 2000, 2001 and 2002 among the employees of the City of Helsinki, Finland, who reached the age of 40, 45, 50, 55 and 60 each year (*N* = 8960, 80% women, 67% responded) [33]. In this study, we used data from the follow-up surveys in Phase 2 in 2007 (*N* = 7332, 83% responded) and Phase 3 in 2012 (*N* = 6814, 79% responded) follow-up surveys. Survey and register data linkages were conducted using the personal identification numbers of participants who had given written consent to such linkage at the time of the survey. The final number of participants who consented to linkages (*N* = 6605), remained employed in 2007 (*N* = 4577) and had full survey information on CMD and covariates was 3560 (Figure 1). The ethical aspects of the HHS have been approved by ethics committees of the Department of Public Health, University of Helsinki, and the City of Helsinki health authorities.

### 2.2. Diagnosis-Specific Sickness Absence

We examined the total number of medically certified and reimbursed diagnosis-specific (ICD-10) and all-cause sickness absence days during the follow-up in 2004–2007. The total number of reimbursed sickness absence days was based on reimbursed sickness allowance spells lasting longer than 10 working days. Sundays and other public holidays are not included in the waiting period for sickness allowance or in the reimbursed days. We examined the two most common diagnostic causes of sickness absence, musculoskeletal diseases (M00–M99) and mental disorders (F00–F99), along with all-cause sickness absence. The sickness absence follow-up period started at the beginning of 2004 and continued until the 2007 survey questionnaire was returned. From the sickness absence days, we extracted any other absences than those due to the employees’ own sickness, such as those due to a child’s sickness. The total number of reimbursed sickness absence days in each diagnostic group during the follow-up period were examined. The numbers of reimbursed sickness absence days in each group were categorised as follows: (1) no sickness absence days, (2) 11–30 days, (3) 31–60 days, and (4) 60 or more days [1]. The first category included participants with no sickness absence days at all as well as those with shorter, non-reimbursed sickness absence spells during the follow-up. Categories 2–4 included participants with various combinations of reimbursed (and non-reimbursed) sickness absence spells. For example, one sickness absence spell of 31–60 days or multiple separate 11-day spells together amounted to 31–60 total days.

### 2.3. Common Mental Disorders

To measure CMD, we used the 12-item version of the Finnish-language version of the General Health Questionnaire (GHQ-12) from the 2007 and 2012 surveys [34,35,36]. The GHQ-12 is a reliable and well-validated questionnaire that measures general and minor mental health problems, such as symptoms of anxiety and depression, and also predicts more severe mental disorders and treatment need [34,35,36]. The 12 items amount to a total score of 0–12, which is commonly dichotomised at points of 1/2, 2/3 or 3/4. In this study, we used the cut-off point of 2/3, which has been used in previous studies of our cohort [25] and has also been recommended for employed populations in general [36]. Thus, participants with a total score of 0–2 were considered a reference group, and participants scoring 3–12 were considered to have CMD. To examine changes in CMD between the two time points of 2007 and 2012, a separate variable indicating change was formed. This CMD change variable consisted of four categories. The “No CMD” category included participants who had no disorders, i.e., a GHQ score of 0–2, at both two time points, i.e., in both 2007 and 2012. The “Favourable change in CMD” category included participants who had disorders in 2007 but not in 2012. The “Unfavourable change in CMD” category included participants who had disorders in 2012 but not in 2007. The category “Repeated CMD” category included participants who had CMD at both time points, i.e., in both 2007 and 2012. 

### 2.4. Covariates

The covariates were from the 2007 survey. The sociodemographic covariates included sex, age and marital status categorised into partnership or no partnership. Socioeconomic position was measured using occupational class derived from the employer’s personnel register and categorised into managers and professionals, semi-professionals, routine non-manual workers and manual workers. Health behaviours included current smoking (yes or no); problem drinking (yes or no) assessed using the CAGE (formed from words Cut-Annoyed-Guilty-Eye in the four questionnaire questions) scale, which is a questionnaire and screening tool for identifying problem drinking and potential alcohol problems [37]; physical activity, categorised into inactive and active on the basis of weekly hours of metabolic equivalent tasks (METs); and body mass index (BMI) based on self-reported height and weight (kg/m^2^) categorised into obese (BMI > 30), overweight (BMI 25–30) and normal (BMI < 25). Work-related covariates included working time (40+ hours per week versus less), shift work versus normal working hours, physical strenuousness measured using a single-item question on the physical heaviness of the respondent’s work, and psychosocial working conditions measured using Karasek’s job strain model in which a combination of high job demands and low job control indicates job strain [38]. 

### 2.5. Statistical Analyses

We examined association of medically certified diagnosis-specific and all-cause sickness absence with subsequent CMD and their changes between two time points with statistical analyses. We first calculated the numbers and percentages for CMD, the covariates, and sickness absence days in each diagnostic group. Second, we fitted the logistic regression models producing odds ratios (OR) with 95% confidence intervals (CI) to examine associations of the total number of sickness absence days in each diagnostic group, i.e., those caused by mental disorders, musculoskeletal diseases and all causes, with subsequent CMD at the two follow-up time points, 2007 and 2012. We also adjusted for age, sex, marital status, occupational class, health behaviours and working conditions. Sex interactions were tested, and as they were not statistically significant, the analyses were conducted using pooled data of women and men. In further models, we used multinomial regression analysis to examine change in CMD as the outcome. First, we fitted age- and sex-adjusted models, after which we added marital status, occupational class, health behaviours and working conditions. The analyses were performed using SAS statistical software, version 9.4 (SAS Institute Inc., Cary, NC, USA).

## 3. Results

Of all the participants, 24% reported CMD in 2007 and 21% in 2012 (Table 1). No CMD was reported by 76% of participants in 2007 and 79% in 2012, whereas 11% had a favourable change from 2007 to 2012, 14% an unfavourable change and 10% had CMD in both 2007 and 2012. Of all the participants, 33% had at least one sickness absence spell of 11 or more working days due to any cause (Table 2). The corresponding figures for sickness absence due to mental disorders and musculoskeletal diseases were 7% and 13%, respectively. The prevalence percentages of CMD, especially of CMD in 2007, were in general higher among participants with sickness absence due to mental disorders or musculoskeletal diseases or any cause. In addition, prevalence percentages of unfavourable change in CMD and repeated CMD were higher overall among participants with sickness absence due to mental disorders, musculoskeletal diseases or any cause. Table 2. Prevalence of diagnosis-specific sickness absence (SA) (*N*, %) and common mental disorders (CMD, % in 2007, 2012 and change).

### 3.1. Associations between Sickness Absence and Subsequent Common Mental Disorders at Two Time Points

Using logistic regression models, we examined the associations between total number of sickness absence days in 2004–2007 due to mental disorders and musculoskeletal diseases and all-cause absence, and subsequent CMD at two time points, in 2007 and 2012 (Table 3). For all-cause sickness absence, age- and sex-adjusted Model 1 showed a gradient, as a higher number of sickness absence days was associated with having CMD in both 2007 and 2012. The highest risk of CMD (2007 OR=3.20, CI 2.59–3.97; 2012 OR: 1.63, CI 1.29–2.05) was found among participants with the highest number (61+ days) of all-cause sickness absence days. For sickness absence due to mental disorders, an even stronger association was observed, as the risk of CMD doubled or tripled in the groups with 11+ sickness absence days in 2007 (OR range: 2.16–2.93) and almost doubled also in 2012 (OR range: 1.81–1.93). For sickness absence due to musculoskeletal diseases, participants with 11-30 SA days were at no higher risk of CMD than the reference group, but for those with 31+ sickness absence days we observed a high risk of CMD in both 2007 (OR range: 2.79–2.93) and 2012 (OR range: 1.72–2.03). Controlling for the covariates in Models 2 and 3 attenuated some of the associations, mainly those with CMD in 2012, but most of the associations remained. 

### 3.2. Associations between Sickness Absence and Change in Subsequent Common Mental Disorders

Using multinomial regression models, we examined the associations between the total number of sickness absence days in 2004–2007 due to mental disorders and musculoskeletal diseases and all-cause absence, and change in subsequent CMD between the two time points of 2007 and 2012 (Table 4). In the age- and sex-adjusted models, having a higher number of all-cause sickness absence days was associated with favourable as well as unfavourable change in CMD, and most strongly with repeated (OR range: OR 1.56, CI 1.10–2.20 – OR 3.28, CI 2.42–4.45) CMD. Similarly, a higher number of sickness absence days due to mental disorders was associated with both favourable change and unfavourable change, and most strongly with repeated (OR range: OR 2.61, CI 1.19–5.72 – OR 3.36, CI 1.83–6.18) CMD. Sickness absence due to musculoskeletal diseases was associated with favourable change in CMD only in the 31–60 SA-day group but with unfavourable change in CMD in all 11+ SA-day groups (OR range: OR 1.53, CI 1.01–2.30 – OR 3.37, CI 2.05–5.53). Moreover, associations with repeated CMD were found for the groups with 31–60 (OR: 3.28, CI 1.86–5.82) and 61+ (OR: 3.40, CI 2.23–5.18) sickness absence days due to musculoskeletal diseases. In the models adjusted for health behaviours, working conditions and other covariates, having a higher number of all-cause sickness absence (SA) days remained associated with unfavourable change and repeated CMD, but the association with favourable change did not remain statistically significant. In the associations between sickness absence due to mental disorders and change in CMD, we observed some attenuation, but the associations mostly remained statistically significant. In the models adjusted for working conditions and other covariates, the associations were very similar to those obtained by adjusting for health behaviours. 

## 4. Discussion

This study examined the associations of diagnosis-specific and all-cause sickness absence with subsequent CMD and their changes between two time points among midlife and ageing employees of the City of Helsinki, Finland. The main results of the study were that all-cause sickness absence, as well as sickness absence due to mental disorders and musculoskeletal diseases, increased the risk of CMD after sickness absence follow-up (2007) as well as five years later (2012). Stronger associations were observed for the more proximal time point of CMD. In addition, all-cause sickness absence as well as absence due to mental disorders and musculoskeletal diseases were all associated with changing and repeated CMD. 

Previous evidence from studies of other European employee populations as well as of our own cohort shows that CMD are associated with an increased risk of subsequent sickness absence due to different diagnostic causes as well as of different durations [22,25,26,27,28,29,30,31,32]. To our knowledge, no previous studies have, however, investigated CMD among employees after medically certified diagnosis-specific or all-cause sickness absence. Some evidence is available of other mental and general health and functioning outcomes, with which our results are in line. This evidence includes results from a study of our own cohort, which showed sickness absence to be associated with subsequent poorer physical and mental functioning measured by the Short Form 36 [1]. In that study, sickness absence due to mental causes was most strongly associated with poor mental functioning and the associations persisted over the five-year survey-based follow-up. Absences due to somatic causes also showed some, although weaker, associations with poorer mental functioning. A Swedish study examining self-reported sickness absence found a higher number of sickness absence days during the past year to be associated with poorer survey-based mental well-being and self-rated health in the following year [6]. Regarding more severe mental health outcomes, other Swedish studies based on the entire non-retired adult population of the country, have shown sickness absence due to both mental and somatic causes to increase the risk of suicidal behaviour, among both women and men, and that it is a risk factor for subsequent inpatient care [7,39]. 

The results of the present study differed to some degree depending on the time of CMD measurement. The first follow-up in 2007 was just after the end of the sickness absence register follow-up period, whereas the second follow-up was five years later in 2012. The associations were notably stronger with more proximal CMD, but they still remained five years later. Furthermore, examination of changes in CMD between those two time points showed both all-cause and diagnosis-specific sickness absence to be especially associated with an unfavourable change in CMD as well as repeated disorders. Somewhat weaker associations were found with favourable change in CMD. The participants in this category had CMD at the first follow-up time point but not at the second, which could mean that favourable change, although better than repeated CMD, is still worse than having no disorders at all. Overall, according to our results, it seems that a high number of medically certified sickness absence days, regardless of diagnostic cause, could increase the risk of poorer or declining general mental health either immediately after sickness absence or even many years later. However, there could also be changes in either direction between the two measuring time points, as CMD may vary over time with changes in the prevalence, composition and severity, including alternation of depression and anxiety type of symptoms especially among ageing adults [40,41,42]. 

Sickness absence due to both mental disorders and musculoskeletal diseases increased the risk of subsequent CMD in this study. Previous results are lacking on these associations, but there is evidence that CMD could increase the risk of subsequent sickness absence and disability retirement due to both mental and somatic causes [24,32]. Other studies have also shown comorbidity or interconnectedness between mental disorders and musculoskeletal diseases, and the results of our study could thus be seen as confirming previous observations regarding relationship between mental and somatic health [43]. 

In this study, due to potential differences between women and men in the CMD for example in their prevalence, we first fitted the base models sex-stratified, which showed slight differences in estimates. However, we found no statistically significant differences between women and men when testing for interactions. There could have been sex differences, for example, in how sickness absence due to the different diagnoses were associated with CMD. Previously, sex-differences have been found in regards to more severe mental health outcomes, as the risk of suicidal behaviour was shown to be higher among Swedish men than women after sickness absence due to mental diagnoses [7]. There could be differences between women and men not only in the prevalence of mental health disorders, but also in reporting them, as well as care-seeking behaviour and identifying the disorders in healthcare. 

Overall, the findings of the present study provide further evidence of the potential negative health consequences of sickness absence, which should be taken into account when planning measures to support employees with longer periods of absence, and their return to work. To promote the return to work of employees with CMD, and to prevent further sickness absence and employee mental health problems in general, different kinds of interventions have been developed and could be applied in work settings [44,45]. In occupational healthcare, case management protocols could benefit employees after long-term sickness absence and should be established to ensure access to care, thus preventing decline in employee mental health and the development of mental disorders. Further research would be needed to clarify potential differences between age and socioeconomic groups in how sickness absence is associated with subsequent mental health. 

The study had several strengths, the first of which was its prospective longitudinal design using a linked register and survey data. The comprehensive data, which included detailed register information concerning medically certified diagnosis-specific sickness absence from national registers, increased the reliability and validity of this study. The second strength was that the data enabled us to compare the different diagnostic sickness absence groups, which is rarely done, thus contributing to research on the possible interrelations between the different domains of health. Finally, the survey information that was available on CMD at the two time points, was based on the well-validated and widely used instrument and screening tool, the GHQ-12. In sensitivity analyses, we were also able to control for prior CMD, also measured by the GHQ-12 at baseline, which we found to have no effects on the observed associations (data not shown). In general, it is likely that the results of this study are, with caution, to some degree generalisable to ageing employees in the municipal sector and possibly elsewhere in the public sector, at least in Finland.

Some limitations should be considered when interpreting the findings of this study. Firstly, the whole study period was long, reaching from the beginning of the sickness absence register follow-up in 2004–2007 to the 2012 survey, a total of approximately eight years. During this time, there may have been variation, such as changes in either direction in both CMD and covariates, that remained unmeasured but might have affected the observed associations. Related to this limitation, the disorders measured by the GHQ-12 were from the last four weeks and this may also have affected the associations, which were followed-up for a long period. In addition, due to the long study period it is possible that selection took place and only the most robust part of the study population could have remained in the data. It is thus plausible to think that even stronger associations may have been seen if the least healthy employees who had already left due to, for example disability retirement, had still been included in the study population. Secondly, as the age group that we studied may have differed from other age groups, and the municipal employees from other employee groups, direct generalisations to all employed populations should be avoided. Thirdly, the survey-based data warrants general caution due to the possibility of under- or over-reporting and non-response. Analyses of the HHS data have shown that the data represents the target population, the employees of the City of Helsinki, to a satisfactory degree, but non-response was slightly more common at baseline among participants with previous sickness absence compared to those without sickness absence [46].

## 5. Conclusions

This study showed that the number of sickness absence days in 2004–2007 due to mental disorders and musculoskeletal diseases, as well as all-cause sickness absence, were associated with subsequent CMD in 2007 and 2012 in a cohort of Finnish municipal employees. The strongest associations were observed with CMD that were more proximal to the sickness absence follow-up. Associations were also found with CMD changes between the two examined time points and especially with repeated CMD. Attention should be paid to the mental health consequences of sickness absence periods due to any diagnostic cause, mental or somatic, in order to support the health of midlife and ageing employees and to prevent further decline in work ability. 

## Figures and Tables

**Table 1:** Distribution of participants (*N*, %) by covariates.

Covariate	Category	N	%
Sex	Women	2904	82
	Men	656	18
Age	45–47	808	23
	50–52	925	26
	55–57	956	27
	60–67	871	25
Marital status	Partnership	2485	70
	No partnership	1075	30
Occupational class	Managers and professionals	1155	32
	Semi-professionals	787	22
	Non-manual employees	1155	32
	Manual workers	463	13
Working time	Less than 40 hours / week	3025	85
	40 hours or more / week	535	15
Shift work	No	2864	80
	Yes	697	20
Physical strenuousness of work	Light	892	25
	Moderate	1610	45
	Strenuous	1058	30
Job strain	No	2803	79
	Yes	757	21
Current smoking	No	2953	83
	Yes	606	17
Problem drinking	No	2828	21
	Yes	732	79
Physical inactivity	No	2972	83
	Yes	588	17
Body mass index	Normal	1754	49
	Overweight	1213	34
	Obese	593	17
All		3560	100

**Table 2:** Prevalence of diagnosis-specific sickness absence (SA) (*N*, %) and common mental disorders (CMD, % in 2007, 2012 and change).

CMD Change 2007–2012 ^a^								
SA by Diagnostic Causes, Total Number			2007	2012	No	Favourable	Unfavourable	Repeated
of Reimbursed Days in 2004–2007	*N*	%	CMD %	CMD %	CMD %	Change %	Change %	CMD %
**All-cause SA days**								
No SA days	2384	67	19	19	70	11	11	8
11–30	432	12	26	22	63	12	15	11
31–60	289	8	31	26	57	13	17	13
61+	455	13	43	27	47	10	26	17
**SA days due to mental disorders**								
No SA days	3304	93	22	20	67	11	13	9
11–30	118	3	42	32	45	13	23	19
31–60	51	1	39	33	45	16	22	18
61+	87	2	46	33	39	15	28	18
**SA days due to musculoskeletal diseases**								
No SA days	3102	87	22	20	67	11	13	9
11–30	181	5	26	22	60	14	18	8
31–60	103	3	44	33	41	16	26	17
61+	174	5	45	29	46	9	25	20
**All**	3560	100	24	21	65	11	14	10

^a^ Favourable change = CMD in 2007 only. Unfavourable change = CMD in 2012 only. Repeated CMD = CMD in both 2007 and 2012.

**Table 3:** Associations between diagnosis-specific sickness absence (SA) and subsequent common mental disorders (CMD, GHQ-12 score 3+), odds ratios (OR) with 95% confidence intervals (CI) from logistic regression models. (*N* = 3560).

Total Number of Reimbursed SA days in 2004–2007	MODEL 1 ^a^		MODEL 2 ^b^		MODEL 3 ^c^	
	CMD, OR (95% CI)		CMD, OR (95% CI)		CMD, OR (95% CI)	
	2007	2012	2007	2012	2007	2012
**All–cause SA**						
No SA days	1.00	1.00	1.00	1.00	1.00	1.00
11–30	1.48 (1.17–1.89)	1.25 (0.97–1.60)	1.48 (1.16–1.89)	1.19 (0.93–1.54)	1.52 (1.19–1.94)	1.24 (0.97–1.60)
31–60	1.85 (1.41–2.43)	1.50 (1.13–1.99)	1.82 (1.38–2.40)	1.42 (1.07–1.90)	1.79 (1.35–2.36)	1.44 (1.08–1.92)
61+	3.20 (2.59–3.97)	1.63 (1.29–2.05)	3.12 (2.50–3.89)	1.49 (1.17–1.89)	3.16 (2.53–3.94)	1.54 (1.21–1.95)
**SA due to mental disorders**						
No SA days	1.00	1.00	1.00	1.00	1.00	1.00
11–30	2.45 (1.68–3.57)	1.81 (1.22–2.70)	2.27 (1.55–3.32)	1.70 (1.13–2.54)	2.31 (1.57–3.39)	1.71 (1.15–2.56)
31–60	2.16 (1.22–3.82)	1.84 (1.02–3.33)	1.85 (1.03–3.31)	1.63 (0.89–2.96)	1.89 (1.06–3.39)	1.69 (0.93–3.08)
61+	2.93 (1.91–4.51)	1.93 (1.22–3.04)	2.53 (1.63–3.93)	1.64 (1.03–2.61)	2.58 (1.66–4.01	1.74 (1.09–2.76)
**SA due to musculoskeletal diseases**						
No SA days	1.00	1.00	1.00	1.00	1.00	1.00
11–30	1.24 (0.88–1.76)	1.14 (0.79–1.63)	1.26 (0.89–1.79)	1.07 (0.74–1.55)	1.23 (0.86–1.75)	1.09 (0.75–1.57)
31–60	2.79 (1.87–4.16)	2.03 (1.33–3.10)	2.83 (1.88–4.26)	1.97 (1.28–3.03)	2.79 (1.85–4.20)	1.93 (1.26–2.97)
61+	2.93 (2.14–4.01)	1.72 (1.22–2.42)	2.96 (2.14–4.09)	1.66 (1.17–2.36)	2.96 (2.14–4.10)	1.64 (1.16–2.33)

^a^ Adjusted for age, sex. ^b^ MODEL 1 + marital status, occupational class, smoking, problem drinking, physical inactivity, BMI. ^c^ MODEL 1 + marital status, occupational class, working time, shift work, physical strenuousness of work, job strain.

**Table 4:** Associations between all-cause sickness absence (SA) and SA due to mental disorders and musculoskeletal diseases and subsequent change in common mental disorders (CMD, 2007-2012), odds ratios (OR) with 95% confidence intervals (CI) from multinomial logistic regression models (N=3560).

	MODEL 1 ^a^			MODEL2 ^b^			MODEL 3 ^c^		
	Change in CMD ^d^ (ref = no CMD)			Change in CMD ^d ^(ref = no CMD)			Change in CMD ^d ^(ref = no CMD)		
Total number of reimbursed SA days in 2004–2007	Favourable	Unfavourable	Repeated	Favourable	Unfavourable	Repeated	Favourable	Unfavourable	Repeated
	Change	Change		Change	Change		Change	Change	
	OR ^d^ (95% CI)	OR ^d^ (95% CI)	OR ^d^ (95% CI)	OR ^d^ (95% CI)	OR ^d^ (95% CI)	OR ^d^ (95% CI)	OR ^d^ (95% CI)	OR ^d^ (95% CI)	OR ^d^ (95% CI)
All–cause SA days									
11–30	1.18 (0.85–1.64)	1.50 (1.11–2.02)	1.56 (1.10–2.20)	1.14 (0.82–1.59)	1.52 (1.12–2.07)	1.49 (1.05–2.11)	1.19 (0.85–1.66)	1.56 (1.15–2.11)	1.56 (1.10–2.22)
31–60	1.43 (0.98–2.09)	1.89 (1.34–2.67)	2.05 (1.40–3.02)	1.38 (0.94–2.02)	1.91 (1.35–2.70)	1.93 (1.30–2.86)	1.41 (0.96–2.06)	1.86 (1.32–2.64)	1.93 (1.30–2.86)
61+	1.41 (1.00–1.99)	3.45 (2.66–4.49)	3.28 (2.42–4.45)	1.33 (0.94–1.89)	2.48 (2.66–4.56)	1.99 (2.18–4.10)	1.40 (0.98–1.98)	3.52 (2.69–4.62)	3.08 (2.25–4.22)
SA due to mental									
disorders									
11–30	1.66 (0.93–2.99)	2.48 (1.54–3.99)	2.98 (1.80–4.96)	1.59 (0.88–2.87)	2.32 (1.44–3.76)	2.70 (1.62–4.52)	1.63 (0.90–2.93)	2.38 (1.47–3.86)	2.74 (1.64–4.59)
31–60	1.99 (0.88–4.48)	2.38 (1.15–4.93)	2.61 (1.19–5.72)	1.80 (0.79–4.09)	2.06 (0.99–4.31)	2.12 (0.95–4.73)	1.84 (0.81–4.19)	2.07 (0.99–4.32)	2.28 (1.03–5.06)
61+	2.23 (1.17–4.28)	3.54 (2.07–6.03)	3.36 (1.83–6.18)	1.94 (1.00–3.75)	3.11 (1.81–5.34)	2.72 (1.46–5.06)	2.11 (1.10–4.05)	3.18 (1.85–5.47)	2.85 (1.53–5.30)
SA due to musculoskeletal								
diseases									
11–30	1.41 (0.90–2.21)	1.53 (1.01–2.30)	1.02 (0.58–1.78)	1.35 (0.85–2.13)	1.60 (1.05–2.42)	0.97 (0.55–1.70)	1.38 (0.88–2.19)	1.56 (1.03–2.37)	0.95 (0.54–1.68)
31–60	2.41 (1.34–4.35)	3.37 (2.05–5.53)	3.28 (1.86–5.81)	2.41 (1.33–4.38)	3.52 (2.13–5.83)	3.21 (1.79–5.74)	2.36 (1.30–4.28)	3.47 (2.09–5.76)	3.15 (1.76–5.65)
61+	1.26 (0.73–2.19)	2.80 (1.90–4.13)	3.40 (2.23–5.18)	1.26 (0.72–2.21)	2.91 (1.95–4.34)	3.29 (2.13–5.09)	1.26 (0.72–2.21)	2.95 (1.97–4.40)	3.24 (2.09–5.03)

^a^ Adjusted for age, sex. ^b^ MODEL 1 + marital status, occupational class, smoking, problem drinking, physical inactivity, BMI. ^c^ MODEL 2 + marital status, occupational class, working time, shift work, physical strenuousness of work, job strain. ^d^ Favourable change = CMD in 2007 only, unfavourable change = CMD in 2012 only, repeated CMD = CMD in both 2007 and 2012.
